# Assessment of Occupational Carcinogenic Risk by Comparing Data from the Italian Register of Occupational Exposures to Carcinogens (SIREP) with the International Agency for Research on Cancer (IARC) Evidence

**DOI:** 10.3390/ijerph20105850

**Published:** 2023-05-17

**Authors:** Lorena Paris, Alberto Scarselli, Alessandro Marinaccio, Stefania Massari

**Affiliations:** Department of Occupational and Environmental Medicine, Epidemiology and Hygiene, National Institute for Insurance against Accident at Work (INAIL), 00143 Rome, Italy

**Keywords:** occupational exposure, registration system, carcinogens, health surveillance, risk assessment

## Abstract

In Italy, the National Register on Occupational Exposure to Carcinogens (SIREP) is established pursuant to article 243 of Legislative Decree 81/2008 and is aimed to collect information on the exposure of workers to carcinogens transmitted by employers. The aim of this study is to assess its level of implementation comparing prevailing carcinogens reported in SIREP with the monitoring of risks in the workplace evidenced by the International Agency for Research on Cancer (IARC). The data reported in the SIREP have been integrated with IARC and the database on carcinogenic risk in the workplace named MATline in order to build a matrix containing the carcinogens classified according to the IARC (Group 1 and 2A agents) and to a semi-quantitative indicator of risk level (High or Low) calculated upon the number of exposures reported in SIREP. The matrix contains the following data: carcinogens, economic sector (NACE Rev2 coding) and cancer sites. The comparison between SIREP and IARC evidence allowed us to highlight situations with a high risk of carcinogenicity and to address appropriate actions of prevention to contain the risks of exposure to carcinogenic substances.

## 1. Introduction

Exposure to occupational carcinogens is a major public health issue, and the monitoring of such exposures is essential for preventing occupational cancer in the workplace [1].

Occupational exposure to carcinogens is one of the leading causes of death, with an estimated occurrence of 349,000 cancer deaths due to occupational carcinogens worldwide [2] and of 120,000 work-related cancers and around 80,000 deaths per year in Europe [3]. An important tool for a more comprehensive analysis of occupational carcinogens is provided by the Global Burden of Disease series of studies that include a summary of updated findings for occupational carcinogenic risk factors at the national and global levels. The study highlights an important role of asbestos, even if its use decreased due to the ban, and the widespread use of diesel engine exhaust, trichloroethylene, silica, polycyclic aromatic hydrocarbons, chromium, benzene and formaldehyde in the industrial setting [4].In Italy, the number of workers exposed to carcinogens, according to CAREX (carcinogen exposure) estimates, is 4.2 million workers, i.e., 24% of the workforce [5]. At a global level, occupational exposure to carcinogens has tended to stay stable, with a slight decrease between 1990 and 2019 [6]. Although these exposures are largely preventable and there is a regulatory requirement to avoid or minimize them within strict limits, the burden of cancer resulting from the exposure of workers to carcinogens is still a problem and a lot needs to be accomplished to deepen the knowledge of the harmful effect of such exposure to workers’ health. The problem can be tackled either by registration systems or by estimates based on ad hoc surveys.

Within the European Union (EU), some countries have national registers of occupational exposure to carcinogens that provide information on the number of workers exposed, as well as the methods and levels of exposure. Many countries have information systems that collect measurement results, while others have information systems to estimate the number of workers exposed to carcinogens and the relative levels of exposure. [7].

In Italy, prevention and safety in the workplace is regulated by the Legislative Decree no. 81/2008 [8], which, implementing some EU directives, establishes that employers must keep a record of exposures to carcinogens to which workers are involved. This register, named SIREP, contains information about the carcinogens that workers are exposed to, including the exposure level (intensity, frequency and duration) and is held by the National Institute for Insurance against Accidents at Work (INAIL) [9]. Carcinogens present in SIREP are substances, mixtures or procedures classified into two categories, 1A (known to be carcinogenic and/or mutagenic to humans) or 1B (presumed to be carcinogenic and/or mutagenic to humans) according to Regulation (EC) no. 1272/2008 [10] and mentioned in Annex XLII to the Legislative Decree no. 626/1994 [11].

The Italian registration system is different from other European registries because it is characterized by a statutory obligation, which concerns all employers, to notify workers when they have exposed to carcinogens. 

The aim of this study is to assess the scenario of the occupational exposure to cancer-causing agents in Italy according to the results of the Information System Registers on Occupational Exposure to Carcinogens (SIREP).

A comparison with IARC evidence, by means of a critical appraisal of the results, will direct setting priorities for monitoring occupational exposures and preventing health issues in specific workers’ group. 

## 2. Materials and Methods

In this study, we compiled a three-way matrix containing information about the association of carcinogenic agents, industrial processes and cancer sites through integration of different sources such as the IARC [12], MATline databases (the database on carcinogenic risk in the workplace) [13] and SIREP.

The IARC provides associations between human cancer and carcinogen agents, and SIREP provides information on occupational exposures by agent and industrial sector. In order to integrate IARC information on target organ–carcinogens and SIREP data on industrial sectors–carcinogens, we need an additional source able to link cancer site, carcinogens and the industrial sector.

For this reason, we queried MATline database (the database on carcinogenic risk in the workplace) [13,14], which provides information on industrial processes combined with the substances considered carcinogenic according to the IARC classification and the associated target organs.

The integration of these databases gave rise to the three-way matrix, which contains following data: cancer sites, economic sector (recoded in the NACE classification [15]) and carcinogen agent. Such a matrix provides information available in scientific literature, which was used to assess state of cancer-causing exposures reported to SIREP registry. To verify the conformity of notification of occupational exposure to carcinogens reported by employers according to indications of carcinogenicity declared by IARC, we classify SIREP data according to risk levels.

In detail, a semi-quantitative indicator (SIREP risk level) was calculated considering for each agent or class of agents (e.g., polycyclic aromatic hydrocarbons (PAHs)) the number of exposures by industrial sector and then evaluating the 25° and 75° percentiles values. 

Since we considered only agents classified by the IARC as group 1 and 2A, SIREP data were sectioned off into 2 classes: High (H) for industrial sectors with a number of exposures greater than the 75° percentile and Low (L) for industrial sectors with a number of exposures lower than the 25° percentile. 

In such way, we standardized SIREP and IARC data in similar groups of risk useful for the comparison.

## 3. Results

The size of the SIREP archive consisted of about 490,000 exposure situations and 223,000 exposed workers up to 31 December 2021. In Table 1, the distribution of exposed workers and exposures by prevailing carcinogens notified in SIREP is presented. The term “exposure” refers to a specific job task of a worker involving exposure to a specific carcinogen or a group of carcinogens (i.e., a group of agents having similar characteristics, for example, the PAH class of hexavalent chromium compounds).

The most frequently reported carcinogen is hardwood dust, followed by benzene and hexavalent chromium compounds.

In Table 2, Table 3 and Table 4, the results of the comparison of prevailing carcinogens reported to SIREP and evidence from the IARC are illustrated in order to monitor carcinogen risk in Italian industrial sectors. These tables show data resulting from the linkage of agents and occupational activities according to IARC risk levels 1 and 2A combined with the SIREP risk levels High and Low. Each carcinogen is listed in descending order, from the most to the least reported agent to the SIREP. In particular, Table 2 shows carcinogen agents with strong evidence from the IARC (Group 1) that are highly reported in SIREP, along with the associated cancer sites and related industrial sectors. This suggests a good level of compliance between notifications and the carcinogenic risk assessment. Examples of such a category include hardwood dust, asbestos and hexavalent chromium. 

Table 3 shows carcinogens with limited evidence from the IARC (Group 2A) that are highly reported in SIREP, along with the associated cancer sites and related industrial sectors. Such categories include benzene, formaldehyde and hexavalent chromium.

Hexavalent chromium is shown in Table 2 and Table 3 as belonging Group 1 and 2A since it is associated with different target organs. 

Table 4 shows carcinogenic agents with strong evidence from the IARC (Group 1) with lower rates of reporting in SIREP, along with the associated cancer sites and related industrial sectors. This category covers many agents such as silica crystalline, formaldehyde and leather dust. This table helps demonstrate in which industrial contexts appropriate prevention and awareness programs in the assessment of carcinogenic risks are required.

Formaldehyde is shown in Table 3 and Table 4 as Group 2A and 1 since it is associated with different target organs. 

## 4. Discussion

Recording occupational exposure to carcinogens is of primary importance not only for the development of a monitoring system of risk factors in the workplace [16] but also for preventing the onset of occupational cancers attributed to exposure to carcinogens. 

The aim of this work is the assessment of carcinogenic occupational risk, resulting from the SIREP, and its critical appraisal, considering the IARC evidence of carcinogenicity for humans.

The estimates from SIREP show that the most reported carcinogens are hardwood dust, benzene, hexavalent chromium and formaldehyde (see Table 1). 

Hardwood dust is present in Italy in the manufacturing of furniture and joinery installation, as well as in the fabrication of machinery equipment and motor vehicles and ship building. All these specific exposures are associated in the literature with sino-nasal and nasopharynx cancer. In the scientific literature, hard heavy wood, derived mostly from deciduous trees, showed high exposure rates in the building of ships and boats and most frequently among woodworking machine setters and operators [17]. In the manufacture of wood products, several cases of exposure to cork dust are found, mainly in the industrial processing of cork and footwear production (Table 2). Italy is a leader in the production, processing and trade of cork, and recently, sino-nasal cancer cases have been observed among subjects occupationally exposed to cork dust [18] and tannins (phenolic compounds found in cork) [19]. 

Exposures to hardwood dust are also shown in the manufacture of leather and related products (Table 4), particularly in tasks concerning the drying operations of fur and leather during skin and fur processing. The use of sawdust is documented in the processing of tanned pelts, which are treated with an oil solution and are then cleaned in rotating drums containing sawdust, which absorbs moisture and excess oil [20]. Moreover, exposure to cork dust was found to be associated with exposure to leather dust in cork sole production and manufacturing [18].

A high number of exposures has been reported in SIREP for the following agents: benzene in association with lympho-hematopoietic and lung cancer and hexavalent chromium and formaldehyde associated with sino-nasal cancer. These agents are classified as probable carcinogens by both the IARC and the EU and, therefore, information derived from SIREP are consistent with the literature.

The myelotoxic action of benzene has been known for decades. In Italy, benzene exposure is regulated by Law no. 245/1963 [21] and Ministerial Decree no. 707/1996 [22], as well as by Ministerial Decree no. 60/2002 [23], which sets as annual limit value on benzene acceptable in urban areas, i.e., 5µg/m^3^. As shown in Table 3, in Italy, benzene is present in the refined petroleum products sector, at petrol stations and in the manufacture of basic chemicals. It is defined by the IARC as a probable carcinogen causing chronic lymphocytic leukemia (CLL), multiple myeloma (MM), non-Hodgkin’s lymphoma (NHL) and lung cancer. In the scientific literature, a high risk is demonstrated for operators of petrol stations breathing benzene [24]. Nowadays, benzene is mainly used in the chemical industry to produce molecules (e.g., styrene, phenol, cyclohexane, quinones) for making insecticides, plastics, paints and drugs [25].

Formaldehyde is widely used in Italian industries, despite being defined by the IARC as a probably carcinogenic to humans associated with sino-nasal cancer, as is hexavalent chromium. 

The strength of the evidence for the carcinogenicity of formaldehyde in association with sino-nasal cancer is quite clear in SIREP for human health activities. However, the association with leukemia, lymphomas and nasopharyngeal cancer does not appear so evident in the agriculture, rubber, chemical and petroleum industries.

Formaldehyde is used mainly in the production of various types of resin that have wide uses as adhesives and binders in the pulp-and-paper industry. Formaldehyde is also used extensively as an intermediate in the manufacturing of industrial chemicals. It is used in agriculture as a preservative for fodder and as disinfectant, too. Formaldehyde’s lower number of exposures have usually been encountered in the rubber industry [26]. In the petroleum industry, formaldehyde is used in large quantities in oil and gas well drilling operations, normally when fracturing the rock and the clay in the subsoil with pressurized water and chemicals in order to release the methane gas trapped in the wells [27]. Short-term exposures to a large amount of the aqueous solution of formaldehyde (formalin), used as a preservative in medical laboratories, an embalming fluid and as a disinfectant, have been reported for pathologists and anatomists in human health and veterinary activities [26,28]. 

A high number of notifications is found in SIREP for workers exposed to hexavalent chromium in the treatment and coating of metals, whereas there are fewer for those in the manufacturing of basic metals. According to the IARC, hexavalent chromium compounds are considered as known carcinogens associated with lung cancer, especially among workers involved in the production of chromates, chromium pigments and electrolyte chromium plating in the treatment and coating metals [29]. The manufacturing of chemical and pharmaceutical preparations is highly reported in epidemiological studies [30], even if the IARC classified it as a probable carcinogen associated with sino-nasal cancer (Table 3). 

In addition to known occupational risks, interesting associations were found among agents with sufficient evidence of carcinogenicity in humans but few exposures reported by Italian companies, e.g., asbestos, silica dust crystalline, leather dust and trichloroethylene. 

These differences could be related to the following reasons: (a) production processes have changed over the years in order to reduce the use of carcinogens and therefore IARC evidence from epidemiological studies differs over time from actual SIREP data; (b) there may be a potential problem of incompleteness of registration in SIREP or different levels of awareness of carcinogenic risks in workplaces by employers. 

The estimates provided by SIREP for many occupational carcinogens are consistent with the scientific literature and with the robustness of the evidence of carcinogenicity for the strength of the association between exposure and target tumors. Our findings suggested a problem of underestimation of exposures: for example, the number of exposures to leather dust as well as crystalline silica appears low while it would be logical to expect the opposite result.

The causal association of leather dust with the development of nasal adenocarcinomas in the boot and shoe industry is widely documented in the scientific literature, and the IARC classifies it as a probable carcinogen, but leather dust exposure is rarely reported in SIREP. The reason for this is due to the criteria of classification used in SIREP, which refer to the EU classification of carcinogenic categories. In fact, leather dust is not classified as carcinogenic by the EU, nor is footwear activity included in Annex XLII. However, in Italian Ministerial Decree no. 81/2008 "New tables of occupational diseases in industry and agriculture", nasal cavity tumors and paranasal sinus tumors in workers manufacturing and repairing footwear are diseases whose denunciation is obligatory since their working origin is of elevated probability.

As far as crystalline silica is concerned, we expect a significant quota of exposed workers in a large variety of industries and occupations due to the extensive natural occurrence of crystalline silica in the Earth’s crust and the use of materials in which it is a component on a large scale.

The main industries and activities liable to expose workers to crystalline silica are construction, in particular the handling or use of sand and other silica-containing products; smelting processes and glass production; and the metal products industry. Since 1997, such findings have been supported by epidemiological results on the association of lung cancer and inhaled crystalline silica (α-quartz and cristobalite) [31] (Table 4). Only with Italian Legislative Decree no. 44/2020 [32] (with which Directive (EU) 2017/2398 was implemented) was the heading “Work involving exposure to respirable crystalline silica powder generated by a treatment process” added in Annex XLII by Legislative Decree 81/2008 (“Activities exposed to cancer-causing agents”). Asbestos is a well-known carcinogen responsible for around 31,572 cases of mesothelioma in Italy, as reported in the Italian National Mesothelioma Register (ReNaM) (period 1993–2018), of which 70% had occupational exposure [33]. In Italy, Law no. 257 of 1992 [34] established the cessation of use and the controlled disposal of asbestos on the basis of its recognized danger to human health and the environment. Since then, the reduced production and use of asbestos has led to a decrease in exposure, especially in the occupational context. Despite this, asbestos continues to be present in workplaces and the environment. Workers involved in the remediation and disposal of asbestos-containing materials (ACMs) continue to be exposed to relevant doses of airborne asbestos fibers, as well as workers employed in economic activities not traditionally associated with asbestos, such as mechanics, plumbers and welders, jewelers and bakers [35]. As shown in Table 2 and Table 4, SIREP collects reports on exposed construction workers, but not workers involved in activities implying an indirect use of asbestos such as the manufacture of motor vehicles and the manufacturing of concrete articles, cement and plaster [33]. The potential exposure to asbestos for construction workers, especially those involved in maintenance and removal of old buildings, is still a real concern in Italy, as discussed in a recent ReNaM epidemiological remark [36].

The low number of notifications of exposure to trichloroethylene (classified as a certain carcinogen by the IARC) may be explained by the decline in the production and use of several of chlorinated solvents since the late 20th century as a result of concerns about their environmental and health effects, including their potential to cause cancer [37]. A confirmation of such data come from the CAREX study, which reported a declining number of workers exposed to trichloroethylene from 42,000 to 34,500 for the years 1990–1993 and 2001, respectively, in Italy [38].

Chlorinated solvents were used in the past to clean and to degrease rollers and other metal parts of printing machines and in textile-furnishing mills, other textile-product mills and plastic product manufacturing [39], but over the years, they have been replaced with tetrachloroethylene and mixtures of soaps and vegetable oils.

In this study, we want to compare the exposures to carcinogenic agents reported in SIREP by force of law, with results deriving from appropriate epidemiological studies published in peer-reviewed journals. This comparison would enable us to classify the carcinogenic properties of an agent. 

On the basis of these studies, the IARC has considered not only individual agents but also mixtures of substances as well as working activities for classification into groups of carcinogenicity, informed by scientific evidence [29]. The activity that the IARC has implemented is aimed at primary prevention that consists of the reduction or elimination of environmental carcinogens. The strength of the evidence suggesting that it induces cancer in humans is evaluated for each agent. This is a qualitative assessment expressed on a scale from the category "carcinogenic" to the "probably carcinogenic" for humans. A quantitative estimate in terms of intensity and duration of exposure is outside the scope of the IARC [40].

The SIREP database, instead, provides data on actual scenarios of occupational exposures to carcinogens. The aim of this register is to promote prevention programs at the workplace to assess, control, eliminate or reduce the number of exposed workers and/or exposures to hazardous agents. The reported results underestimate the number of workers exposed to carcinogens in Italy, as evidenced by CAREX estimates. This is mainly due to the non-compliance with the directive and to the time and resources required to collect all the information contained in the register. Another reason for which SIREP data are underestimated may be the difficulty in determining the inclusion criteria, particularly when the exposures number is low or the amount of the carcinogens used is small. It should also be noted that a worker may have been exposed to more agents at the same time for the same task [2]. Finally, exposure notifications mainly come from large companies, while the number exposures in small companies is underestimated.

## 5. Conclusions

SIREP proved to be a valid tool to monitor occupational exposure to carcinogens and to support prevention activities. Moreover, it has proven to also be useful for research and epidemiological surveillance purposes by facilitating the identification of occupational carcinogens currently used in industrial processes and addressing appropriate actions of prevention in order to contain the risks of exposure to certain or possibly carcinogenic substances.

The comparison of SIREP data with the carcinogenic risk situations known from the literature showed a potential incompleteness in exposure recording. It is reasonable that there is a lack of thorough knowledge, information, awareness and liability related to the use of hazardous substances. To ensure a complete monitoring process and the protection of workers’ health, it should be suitable to plan information and awareness-raising campaigns targeted to employers and employees. The regular collection of data on key carcinogens in the main industrial sectors will provide a valuable set of information on the number of exposed workers and their exposure levels. 

However, the critical assessment of the SIREP enables us to evaluate the effectiveness and ongoing adaptation and improvement of the registry’s design and activities.

## Figures and Tables

**Table 1:** Distribution of prevalent carcinogen agents as a percentage of exposed workers and exposures in SIREP up to 31 December 2021 *.

Carcinogenic Agent	Exposed Workers (%)	Number of Exposures (%)
Hardwood dust	33.2	17.7
Benzene	15.2	9.3
Hexavalent chromium	10.0	6.6
Formaldehyde	6.8	3.3
Nickel compounds	5.8	4.5
Polycyclic aromatic hydrocarbons (PAHs)	5.6	11.1
1,3-Butadiene	4.8	3.0
Silica dust, crystalline	3.5	1.8
Asbestos	3.2	3.3
1,2-Dichloroethane	2.8	1.9
Acrylonitrile	2.5	1.5
Cobalt and cobalt compounds	2.0	1.3
Acrylamide	1.7	0.9
Mineral oils	1.7	1.3
Vinyl chloride	1.7	1.2
Cadmium and cadmium compounds	1.5	0.9
Aromatic amines	1.4	1.1
Trichlorethylene	1.3	0.9
Ethylene oxide	1.1	0.6
Antineoplastic agents	1.0	2.9
Epichlorohydrin	1.0	0.5

* The table shows agents with exposed workers higher than 1%.

**Table 2:** Agents, assessed as Group 1 by IARC and High in SIREP, industrial sector and cancer sites.

Agent	Industrial Sector (NACE Code) ^1^	Cancer Sites
Hard wood dust	Building of ships and boat (30.1)	Sino-nasal
	Manufacture of wood products (16.2)	
Asbestos	Construction (41)	Larynx, Ovary, Pleura, Mesothelioma of pleura and peritoneum, Lung
Mineral oils	Manufacture of refined petroleum products (19.20)	Skin
Hexavalent chromium	Treatment and coating of metals (25.61)	Lung
1,3-Butadiene	Manufacture of rubber products (22.1)	All Lympho-Hematopoietic organs/tissues
Vinyl chloride	Manufacture of refined petroleum products (19.20)	Liver

^1^ Classification according to NACE rev.2.

**Table 3:** Agents assessed as Group 2A by IARC and High in SIREP, occupational activities and cancer sites.

Agent	Industrial Sector (NACE Code) ^1^	Cancer Sites
Benzene	Retail sale of automotive fuel in specialized stores (47.3)	CLL, MM, NHL ^2^, Lung
	Manufacture of basic chemicals (20.1)	
	Manufacture of refined petroleum products (19.20)	
Formaldehyde	Human health and veterinary activities (86)	Sino-nasal
Hexavalent chromium	Manufacture of basic chemicals (20.1)	Sino-nasal
	Manufacture of pharmaceutical products and preparations (21)	

^1^ Classification according to NACE rev.2. ^2^ CLL: Chronic Lymphocytic Leukemia, MM: Multiple Myeloma, NHL: Non-Hodgkin’s Lymphoma.

**Table 4:** Agents assessed as Group 1 by IARC and Low in SIREP, industrial sector and cancer sites.

Agent	Industrial Sector (NACE Code) ^1^	Cancer Sites
Asbestos	Manufacture of motor vehicles, trailers and semi-trailers (29)	Larynx, Ovary, Pleura, Mesothelioma of pleura and peritoneum, Lung
	Manufacture of concrete articles, cement and plaster (23.6)	
Hexavalent chromium	Manufacture of paper and paper products (17)	Lung
	Manufacture of basic metals (24)	
Silica dust, crystalline	Manufacture of furniture in metallic material, safes, armored cabinets, safety locks and padlocks, chandeliers, prams and strollers for children, light alloy frames (31.01)	Lung
	Construction of roads and motorways (42.11)	
	Manufacture of basic metals (24)	
	Manufacture of glass and glass products (23.1)	
Nickel	Manufacture of paper and paper products (17)	Sino-nasal, Lung
	Manufacture of domestic appliances (27.50)	Lung
	Manufacture of medical and dental instruments and supplies (32.50)	
Hardwood dust	Tanning and dressing of leather (15.11)	Sino-nasal, Nasopharynx
Aflatoxins	Manufacture of food products (10)	Liver
Cadmium	Manufacture of ceramic products (23.3)	Lung, Prostate
	Manufacture of essences and perfumes without distillation (23.42)	
	Manufacture of rubber products (22.1)	
Formaldehyde	Agriculture (01)	Leukemias, Lymphomas, Nasopharynx
	Manufacture of paper and paper products (17)	
	Preparation of sensitized materials (20.5)	
	Manufacture of synthetic rubber (20.17)	
	Manufacture of refined petroleum products (19.2)	
Mineral oils	Manufacture of railway locomotives and rolling stock (30.2)	Skin
	Construction (41)	
	Manufacture of bricks, tiles and other building products (23.32)	
Arsenic	Manufacture of wine from grape (11.02)	Prostate, Bladder, Skin, Lung
	Manufacture of glass and glass products (23.1)	
Coal tar pitch	Electrical, plumbing and other construction installation activities (43.2)	Skin, Lung
Leather dust	Manufacture of leather and related products (15)	Sino-nasal
Ortho-Toluidine	Manufacture of synthetic rubber (20.17)	Bladder
Trichlorethylene	Printing and service activities related to printing (18.1)	Kidney

^1^ Classification according to NACE rev.2.

## Data Availability

Not applicable.
